# The Treatment of Abscesses

**Published:** 1893-05-13

**Authors:** 


### THE LONDON HOSPITAL.

### The Treatment of Abscesses.

The causes of abscesses being manifold, the treatment necessarily varies accordingly. But in what may be termed a simple abscess, where there is no very extensive primary lesion of some organ or part of the body as its cause, there are several variations in treatment that may be carried out with benefit, in order to obtain a quick cure of the abscess, and a return of the affected parts to a condition of health. In a small simple abscess, containing only a few teaspoonfuls of pus, fairly superficial, perhaps already thinning the skin over it, an incision long enough to let out the contained pus freely, and then the application of moist heat in some form, is sufficient to effect a cure speedily and safely.

The ways of applying moist heat may be practically reduced to two, poultices and fomentations. The poultice in the treatment of abscess is now entirely discarded in this hospital, being much inferior in every respect to the boracic fomentations now in use. These are made either from specially-prepared boracic lint (lint soaked in a saturated solution of boracic acid and dried), or lint soaked at the time of using in hot boracic solution, wrung almost dry, applied two or three folds thick over the affected part, covered with a larger piece of mackintosh or pink jaconet, then a layer of cotton wool, and the whole kept in place with a bandage.

In many cases of abscess, especially those in connection with glands or bones, evidence of tubercular disease in some form, either in the patient himself or the near relatives, is carefully inquired for, as a history of tubercular affection materially modifies the prognosis as to time of cure. Taking a medium-sized or large abscess, usually subcutaneous at one point, perhaps under or between the layers of muscles, acute, painful, and associated with a certain amount of fever, drainage as well as incision is necessary. This, if the abscess is deep, is performed according to Hilton's method. A skin incision is made, if possible over the most dependent part of the abscess, in order to get good drainage; a director is then pushed into the abscess, the points of a pair of dressing forceps slipped along the groove of the director, the blades separated by opening the handles till an aperture sufficient to allow the finger to be introduced is made, and the pus evacuated. If necessary for the purpose of drainage, a counteropening is made, the abscess cavity is washed out with carbolic lotion (1-40), a piece of perforated drainage tube passed into the cavity, and either dry antiseptic dressings, or, if there is much redness and fever, fomentations applied till the discharge stops; if there be much discharge, the abscess is gently syringed out daily with some weak, warm, antiseptic solution, distention of the cavity with fluid being carefully avoided. If there is not much pyrexia, and the abscess be rather more chronic, an attempt is sometimes made to secure primary union of the abscess walls. In order to obtain this it is an absolute necessity that the pyogenic, or pus forming layer of tissue, forming the abscess walls be removed completely. This is done, of course, under an anæsthetic, first by scraping the walls well with a Volkman's spoon, and frequent washing out with antiseptic lotion, and finally scrubbing the walls with small pieces of wet Gamgee or lint fixed in Spencer Wells' pressure forceps, till the walls have completely lost the slimy feel of the pyogenic membrane, washing out all the debris, injecting a few drams of iodoform emulsion or insufflating some iodoform in powder. If the cavity is large a piece of drainage tube is put in, and firm elastic pressure applied by means of a layer of antiseptic gauze, then cotton wool and an elastic, domette, or flannel bandage, the dressings being so arranged, if possible, that the greatest pressure is put on the part of the abscess cavity furthest from the opening, and being sufficient to bring the walls of the cavity into apposition. If there is no rise of temperature or formation of pus, the dressings are not touched for three days; then the tube, if one has been used, is removed, and elastic pressure put on again for a few days, when firm union of the walls will have taken place. In abscesses of a tubercular nature this plan is used with great success, provided the situation of the abscess be such as to allow pressure to be applied, the greatest care being taken to get rid of every trace of caseous material or lining tissue of the cavity, and iodoform being used more freely than in ordinary cases.

Iodoform, having apparently some definite effect in stopping the formation of pus of tubercular origin, is sometimes used without making an incision into the abscess.

The abscess is emptied by means of a Dieulafoy's aspirator, and a quantity of iodoform in emulsion injected by means of the aspirator. The abscess swells up again to nearly its original size, and then in successful cases, slowly subsides. This method is used in cases where, either from the position and extent of the abscess or the general bad condition of the patient, it is not considered advisable to open the abscess in the usual way.

For large, cold abscesses, more particularly those of tubercular origin, this plan is specially useful, giving the cavity time to contract somewhat should incision eventually become necessary; and in those cases where the pus is absorbed, avoiding the danger that exists of acute tuberculosis following on the opening of a large tubercular abscess, an unfortunate complication occasionally met with.

The iodoform emulsion used is a mixture of 10 to 15 grains of iodoform to an ounce of equal parts of mucilage and glycerine.

Iodoform, in bougies, containing 5 to 10 grains in each, is also used, to hasten the healing of the sinuses occasionally left after the contraction and closure of the greater part of an abscess cavity.

The sinus, after having been scraped out with a sharp Volkman's spoon, has an iodoform bougie inserted into it daily, or twice daily, or it is swabbed out with a solution of nitrate of silver 5 to 10 grains to the ounce. If these measures fail, the sinus is slit up, scraped, and allowed to granulate up from the bottom; boracic fomentations being first applied, then some weak astringent lotion, such as nitrate of silver 2 to 4 grains to the ounce, or sulphate of zinc of the same strength.

In a few cases where the contents of an abscess are unusually fœtid, wood wool wrung out of hot water and sprinkled with chlorinated soda lotion is used instead of boracic fomentations, appearing to have greater effect in removing or counteracting objectionable odours and being equally efficient as a dressing.

